# Emerging strategies in radiation therapy: promises and challenges of spatial fractionation, ultra-high dose rates, and nanoparticles

**DOI:** 10.1088/1361-6463/ae0e2d

**Published:** 2025-10-13

**Authors:** Hamid Ghaznavi, Mohammad Rezaee, Francisco Reynoso, Arash Darafsheh

**Affiliations:** 1 Department of Radiation Oncology, WashU Medicine, St. Louis, MO 63110, United States of America; 2 Department of Radiation Oncology and Molecular Radiation Sciences, Johns Hopkins University, Baltimore, MD 21231, United States of America; 3 Varian Medical Systems, Palo Alto, CA 94304, United States of America

**Keywords:** radiation therapy, spatially fractionated radiation therapy, ultra-high dose rate, FLASH, nanoparticle, dosimetry

## Abstract

Radiation therapy (RT) employs ionizing radiation to kill cancerous cells. However, delivering radiation to tumors, typically embedded within normal tissues, inevitably exposes healthy organs to radiation, leading to collateral damage. This creates a tradeoff between the tumor control probability and normal tissue complication probability, ultimately limiting the dose that can be safely administered. While highly conformal RT techniques have improved tumor targeting and treatment efficacy, they remain inadequate for treating large and radioresistant tumors, pointing out the need for alternative strategies. Spatially fractionated RT, ultra-high dose rate RT, and nanoparticle-enhanced RT are emerging techniques with promise in enhancing tumor control while minimizing normal tissue toxicity. Successful clinical translation of these advanced techniques requires cross-disciplinary efforts aimed at technological innovation, a deeper understanding of the underlying radiobiological mechanisms, and the development of early-phase clinical trials. This paper provides an overview of these techniques and their associated challenges and opportunities.

## 1. Introduction

Cancer remains one of the leading causes of death in the developed world [1]. The primary treatments for cancer include surgery, radiation therapy (RT), and chemotherapy [2]. Other treatments for cancer include stem cell transplant [3], hormonal therapy [4], immunotherapy [5], radiopharmaceutical therapy [6], monoclonal antibodies [7], hyperthermia [8], and photodynamic therapy [9]. A combination of these techniques may be required for treatment with approximately half of cancer patients benefiting from RT [10]. In RT, ionizing radiation (photons, electrons, protons, etc.) is used to kill cancer cells through radiation-induced DNA damage that leads to apoptosis or mitotic death [11]. Radiation dose is defined as the amount of energy absorbed per unit mass of tissue (J Kg^−1^ or Gy, Gray) [12]. The probability of DNA cell survival decreases as radiation dose increases. This dose-dependent relationship forms the basis of radiation oncology dose prescription. Radiation oncologists prescribe the dose to the treatment target (e.g. the tumor) while carefully considering the tolerance doses of neighboring healthy tissues, the so-called organs-at-rick (OARs). Cancer cells are generally more sensitive to radiation than normal cells because they tend to divide more rapidly and have impaired repair mechanisms. However, radiation-induced damage is not selective to tumor cells, and a dose that can be safely delivered to the target is limited by the sensitivity of surrounding normal cells. The balance between tumor cell killing (tumor control probability, TCP) and acceptable radiation-induced side effects (normal tissue complication probability, NTCP) is quantified by the therapeutic ratio [13, 14]. Recent advancements in medical imaging and RT have enabled highly conformal dose delivery which has allowed tumor dose escalation and improved the therapeutic ratio for many tumors. However, effectively treating radioresistant tumors near critical structures still face the challenges imposed by the NTCP. This remains a significant obstacle in contemporary conformal RT (CRT), despite the existing advanced treatment methods [15].

Temporal fractionation is one of the earliest methods to enhance the therapeutic ratio, where the total dose is delivered in multiple fractions allowing a differentiable repair in normal cells compared to tumor cells [13]. Common dose fractionation schemes involve daily doses of 2 Gy (e.g. 60 Gy in thirty 2 Gy-fractions) but may also be delivered in a hyperfractionated approach of smaller doses given more than once a day (e.g. 45 Gy in 30 twice daily fractions), or higher prescription doses delivered in one to five fractions (e.g. 50 Gy in 5 fractions). The size of the dose-per-fraction may impose stricter requirements for dose delivery with high doses per fraction requiring highly conformal dose distributions, advanced imaging and delivery techniques, robust immobilization, and motion management techniques (e.g. stereotactic body RT (SBRT) or stereotactic radiosurgery). These conventional RT approaches are often less effective for advanced-stage cancers, large radioresistant tumors, recurrent cancers, extensive brain tumors, and certain pediatric malignancies [16]. Spatially fractionated RT (SFRT), ultra-high dose rate (UHDR) RT, also known as FLASH RT, and nanoparticle (NP)-enhanced RT are techniques with potential to enhance the therapeutic ratio.

Despite advancements in image-guided RT, intensity modulated RT (IMRT) [17] and volumetric modulated arc therapy (VMAT) [18, 19], as well as adaptive RT [20, 21], radiation-induced toxicity to nearby healthy tissues remains a significant constraint. This constraint limits the maximum dose that can be safely delivered to tumors.

SFRT delivers radiation in a non-uniform pattern of high-dose regions within the tumor that can be particularly effective in large radioresistant tumors [22]. This technique can be delivered using a two-dimensional (2D) GRID dose pattern or a more advanced three-dimensional (3D) lattice dose pattern. These techniques are characterized by their highly heterogenous pattern of peaks of concentrated dose and surrounding lower dose valleys as opposed to the conventional uniform dose distribution.

FLASH RT delivers doses at extremely high dose rates, typically above 40 Gy s^−1^, compared to conventional dose rates of <0.5 Gy s^−1^. This rapid delivery has been shown to selectively reduce normal tissue damage while maintaining effective tumor control in preclinical studies [23].

Innovative strategies that enhance tumor radiosensitivity through a combination of radiation with drugs has also been explored. This approach has now been studied using metallic nanomaterials that enhance radiation dose absorption and coined NP-aided RT. NPs are promising tools in medicine due to their biocompatibility and ability to target cancer cells actively and passively [24]. Their small size (<100 nm) enables accumulation in tumors via the enhanced permeability and retention (EPR) effect [25]. Their high surface area also supports drug delivery and unique optical properties enabling various biomedical applications such as imaging, therapy, and diagnostics. These capabilities make NPs a powerful tool for addressing current treatment limitations [26].

This paper provides an overview of SFRT, FLASH RT, and NP-enhanced RT to provide context for scholars outside of the field of radiation oncology. It discusses the rationale of studying each of these techniques, the current status of research in each field, as well as combination strategies of these techniques.

## 2. RT

**Figure 1.**
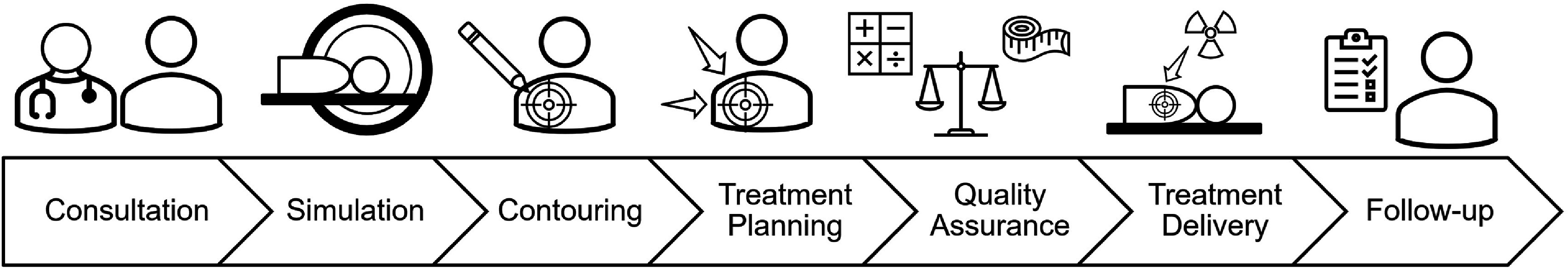
A RT workflow involves initial consultation with the radiation oncologist, obtaining patient’s anatomical information typically through CT scan, and/or MRI and PET, delineating treatment target and organs at risk, treatment planning typically through the treatment planning system, quality assurance either by performing secondary dose calculation or delivering the radiation to phantoms and comparing the measured dose distribution to the calculated one, radiation delivery to the patient, and follow-up visits.

The workflow of conventional RT is shown in figure 1. After consultation with a radiation oncologist, the patient’s anatomy is acquired through a computed tomography (CT) scan and/or other 3D imaging modalities, such as magnetic resonance imaging (MRI) and/or positron emission tomography (PET). The radiation oncologist prescribes the radiation dose (and fractionation scheme) to the target and dose limits to the OARs. Anatomical structures, including the treatment target (e.g. tumor) and the nearby OARs, are delineated on the image set. A treatment planning system (TPS) is used by dosimetrists and/or medical physicists to calculate the dose and obtain an optimized plan to be approved by the radiation oncologist and clinical physicist. Prior to delivery of the radiation to the patient, as a quality assurance (QA) step, the beam is delivered under a measurement setup to compare the measured dose distribution to the TPS calculated one. The patient receives the prescribed dose based on the fractionation scheme and would have follow-up visits during and after completion of the course of RT.

**Figure 2.**
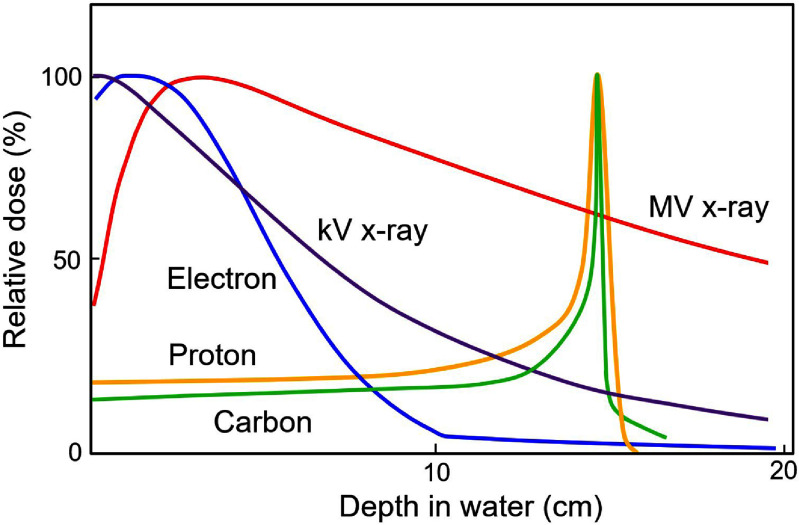
Representative depth dose profiles of radiation beams: ∼200 kVp x-ray, ∼10 MeV x-ray, ∼20 MeV electron, pristine Bragg-peak proton (∼150 MeV), and carbon ion beams (∼290 MeV/nucleon).

External beam RT (EBRT) employs x-ray, electron, and ion beams [12]. A majority of the EBRT beams are delivered using megavoltage (MV) x-ray beams (∼6–18 MV) produced by linear accelerators (linacs) [27]. Figure 2 shows the depth dose distribution in water obtained by different beam types. MV x-ray beams deposit the maximum dose within a few centimeters of tissue beyond which the dose is exponentially reduced by several percentages per centimeter. To spare healthy tissue while achieving a conformal uniform dose distribution within the target, multiple photon beams are typically used at different angles through techniques known as IMRT [17] and VMAT [18, 19].

Electrons are charged particles with a finite range in tissue [27]. Due to their scattering properties, they are not optimal for deep-seated targets and typically used for superficial targets, within a few centimeters from the skin. Typical energies of electrons used in RT are ∼4–20 MeV with a practical range of ∼2–10 cm. The depth into tissue corresponding to 90% of the maximum dose is usually within ∼1–5 cm and corresponds to the clinically meaningful range of tissues that are typically treated. Many linacs commonly used to deliver MV photon beams have the ability to produce therapeutic electron beams.

Ion beams, such as protons and carbons, can penetrate deep into tissue and characteristically deposit the dose by initially losing energy slowly, but as the particles slow down, a majority of the dose is deposited at a certain depth depending on their initial energy [28–30]. Their depth dose profiles show a distinct peak known as the Bragg peak (BP) at the distal end of its range, where most of the dose is deposited with almost no dose beyond that point. As such, ion beams can significantly spare OARs at depths beyond the tumor’s location. However, a single BP cannot cover the tumor’s extent in direction along the beam and a series of BPs with reducing energies and fluences are used to create the so-called spread-out BP to cover the entire depth of the tumor. Proton therapy accelerators include isochronous cyclotrons, synchrocyclotrons, and synchrotrons [31, 32].

**Figure 3.**
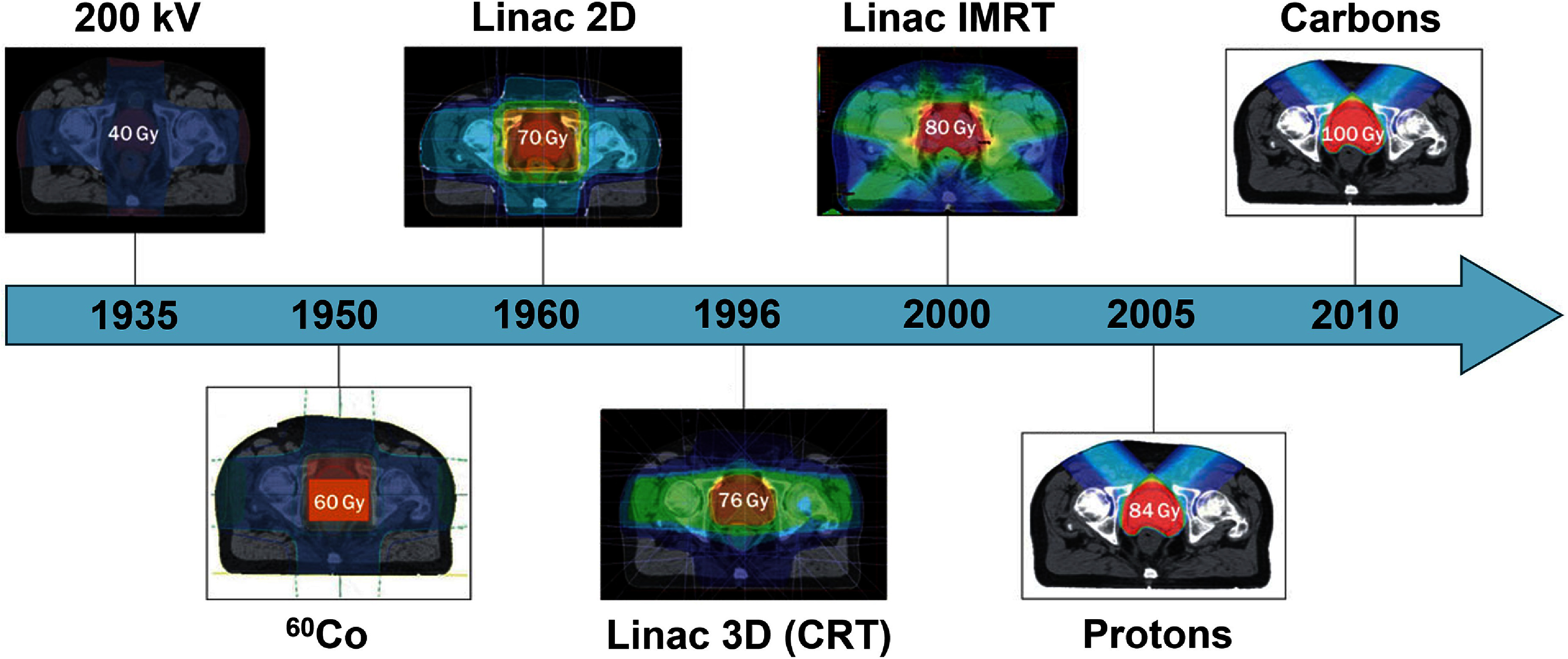
Advances in RT over the past century exemplified for prostate cancer irradiation. Increasing the beam energy and targeting precision allowed dose escalation of the prostate without exceeding the dose limit of healthy tissues allowing transition from palliative RT to curative RT. RT, radiation therapy; 3D-CRT, 3D conformal RT; IMRT, intensity modulated RT. Reproduced from [33], with permission from Springer Nature.

Advances in RT beam delivery techniques have progressed significantly over the last century (figure 3 [33]). During the initial period of RT development (1930’s), lower energy kilovoltage x-ray beams were used to irradiate treatment targets. Due to their poor penetration (high attenuation) in tissue, the total dose to the target was limited by the skin dose or other OAR’s dose tolerance. Invention of ^60^Co RT delivery systems in the 1950’s allowed dose escalation thanks to the high penetration and energy of photons (1.17 MeV and 1.3 MeV) produced in the radioactive decay of ^60^Co. Development of clinical linear accelerators in the 1960’s allowed employing even higher energy x-ray beams to the current practical photon energy range used today (6–18 MV) and furthering dose escalation of the targets. The invention of CT systems allowed precise targeting of the tumors and sparing the OARs through a technique now known as 3D CRT. Development of multileaf collimators (MLCs) allowed fluence modulation and more conformal dose delivery to the target and better sparing of OARs through IMRT and VMAT techniques around the early 21st century. Ion beam therapies gained popularity during the turn of the millennium with currently over 100 proton therapy centers operating around the world. Proton therapy has also emulated photon modulation techniques where multiple x-ray fields at various angles are used to provide a conformal plan through IMRT and VMAT techniques. A similar concept has been implemented in proton therapy to provide a conformal plan through intensity modulated proton therapy [34].

Modern advancements in RT have traditionally been aimed at improving the therapeutic ratio with technological advancements of delivery devices, but several limitations remain. There are biological limitations in terms of normal tissue toxicity, radioresistance, hypoxia, and genetic differences that result in variable response to radiotherapy. There are technological limitations in terms of imaging accuracy and motion management that hinder the ability to deliver high doses to the target while sparing surrounding structures. SFRT, FLASH RT, and NP-enhanced RT have emerged as promising developments with potential to improve on these limitations and enhance RT.

## 3. SFRT

**Figure 4.**
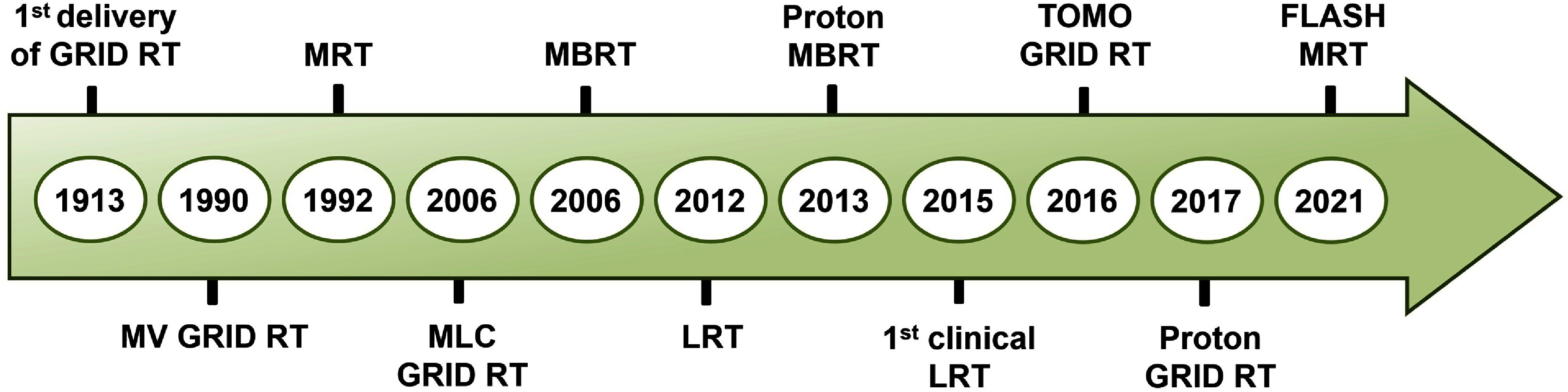
Illustration of the timeline of key milestones in the evolution of SFRT. The field originated with the first delivery of GRID RT by Köhler [37, 38]. Major advancements include the introduction of Megavoltage (MV) GRID RT by Mohiuddin *et al* [39], microbeam radiation therapy (MRT) by Slatkin *et al* [40], development of Multi-Leaf Collimator (MLC) GRID RT by Ha *et al* [41], and minibeam radiation therapy (MBRT) by Dilmanian *et al* [42]. Wu *et al*. implemented the concept of Lattice Radiation Therapy (LRT) [43], followed by the development of proton minibeam radiation therapy by Prezado and Fois [44]. The first clinical report of LRT was published by Suarez *et al* [45]. Further innovations included Tomotherapy GRID (TOMO GRID) RT by Zhang *et al* [46], Proton GRID RT by Henry *et al* [47], and FLASH MRT by Wright *et al* [48].

SFRT has a longer history compared to UHDR and NP-enhanced RTs. SFRT is the delivery of spatially non-uniform dose to a target by segmenting the radiation field into narrow beamlets separated apart spatially, as opposed to conventional RT in which a uniform dose is delivered across the treatment target. SFRT was initially developed in the early 1900s to reduce skin toxicity in cancer patients receiving high doses of orthovoltage (∼200–500 kVp) x-rays. Since then, it has evolved into a clinical strategy for shrinking large deep-seated tumors when uniform dosing would harm normal structures [35]. Over the past thirty years, research has focused on SFRT’s unique biological effects, exploiting tumor cell heterogeneity, differential oxygenation (hypoxic vs normoxic regions), and immune-modulating properties, to achieve a higher therapeutic index than conventional uniform-dose radiotherapy [36]. Figure 4 illustrates the timeline of key milestones in the evolution of SFRT.

The exact mechanism responsible for SFRT is yet to be fully understood. However, various mechanisms have been suggested to play a role in the effectiveness of the SFRT. High-dose radiation in SFRT can induce abscopal effects, triggering immune-mediated systemic responses at distant non-irradiated sites [49, 50]. SFRT also appears to enhance infiltration of immune cells, such as T cells, B cells, and natural killer cells, into the tumor which shows promise in combination with immunotherapy to improve treatment efficacy [51]. Another important effect of SFRT is on the tumor’s blood vessels. High radiation doses (over ∼8–10 Gy) can induce irreversible damage to the blood vessels inside the tumor, making them collapse or change structure, while damage to normal tissue vessels can mostly be repaired [50, 52]. Interestingly, SFRT seems to affect even the parts of the tumor that did not receive a high radiation dose. These bystander effects include signs of immune activation and increased DNA repair activity in the lower-dose regions between the high-dose areas [53, 54]. Additionally, stem cell migration and proliferation in low-dose valleys contribute to normal tissue tolerance, facilitating repair in high-dose regions, as seen in rapid skin regeneration and germ cell survival [55, 56].

**Figure 5.**
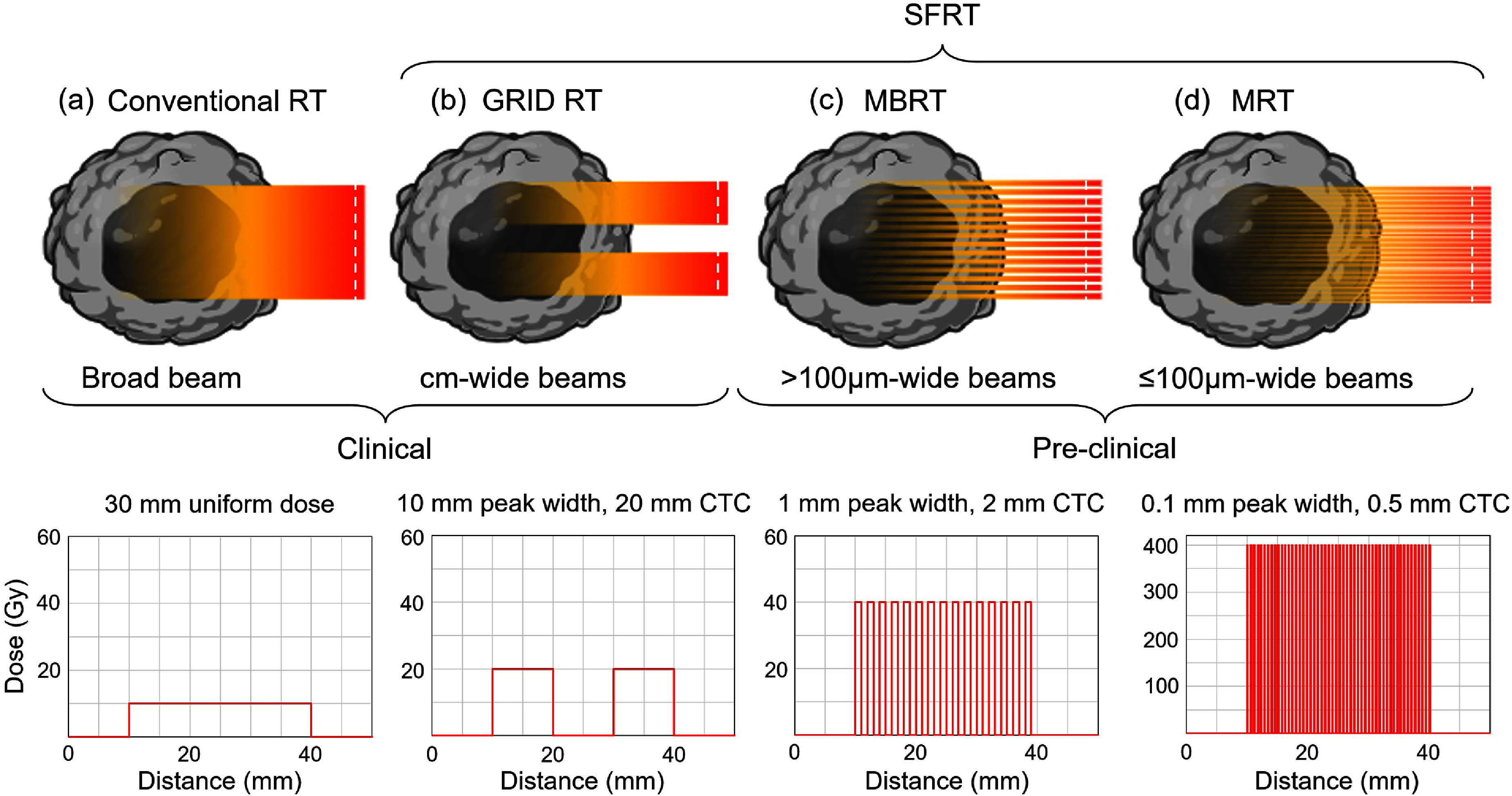
Schematic representation of various 2D SFRT delivery methods with typical cross-beam profiles for peaks and valleys at the collimator surface (white dashed lines) to cover a 30 mm-wide target (black region). The peak dose increases with decreasing aperture size. These profiles degrade with depth into the tissue, depending on the radiation type, energy, and source structure.

Broadly, SFRT can be categorized based on its beam delivery technique as 2D or 3D SFRT. 2D SFRT, also known as GRID RT, delivers radiation with alternating regions of high (peak) and low (valley) doses, arranged in a regular pattern across the treatment field [22]. This is typically achieved using physical collimators with circular or slit apertures. GRID RT includes clinical GRID RT, microbeam RT (MRT), and mini-beam RT (MBRT), depending on the size of the beam shaping apparatus [57, 58]. These 2D SFRT techniques are characterized by parameters of peak dose, valley dose, peak-to-valley dose ratio (PVDR), and the center-to-center (CTC) spacing between apertures. The PVDR is defined as the ratio between the dose delivered in the high-dose regions (peaks) and the adjacent low-dose regions (valleys) created by the beam modulation pattern. The valley dose refers to the minimum absorbed dose in the low-dose regions between peaks, which plays a critical role in normal tissue sparing [57]. Figure 5 provides a schematic overview of dose delivery in different SFRT techniques. 3D SFRT, also called lattice RT (LRT), creates peak and valley dose regions within the tumor volume by utilizing multiple photon beams or the BP of scanning proton pencil beams. Table 1 summarizes important characteristics of these SFRT techniques.

**Table 1.** Characteristics of spatially fractionated radiation therapy (SFRT) techniques reported in the literature.

Feature	Lattice RT	Clinical GRID RT	MBRT	MRT
Beam size	0.5–2 cm	0.5–2 cm	0.2–1.0 mm	20–200 *µ*m
Beam spacing	1–4 cm	1–4 cm	0.8–4 mm	100–800 *µ*m
Radiation type	MV x-rays	Orthovoltage x-rays	Orthovoltage x-rays	Orthovoltage x-rays
	High energy protons	MV x-rays	High energy protons	
		High energy protons		
		High energy ions (C)		
Delivery method	IMRT and VMATPencil beam scanniing	Physical collimators Pencil beam scanning	Physical collimators Pencil beam scanning	Physical collimators
Peak dose (Gy)	5–25	10–20	15–100	> 100
PVDR	Low: $ \unicode{x2A7D} $ 2	Low: $ \unicode{x2A7D} $ 2	Medium: 2–20	High: > 20
Main treatment purpose	Palliative treatment, Reducing normal tissue toxicity,	Palliative treatment, Reducing normal tissue toxicity,	Curative treatment—enhanced therapeutic ratio, Mechanistic studies	Curative treatment—enhanced therapeutic ratio, Mechanistic studies

### 3.1. Clinical SFRT

**Figure 6.**
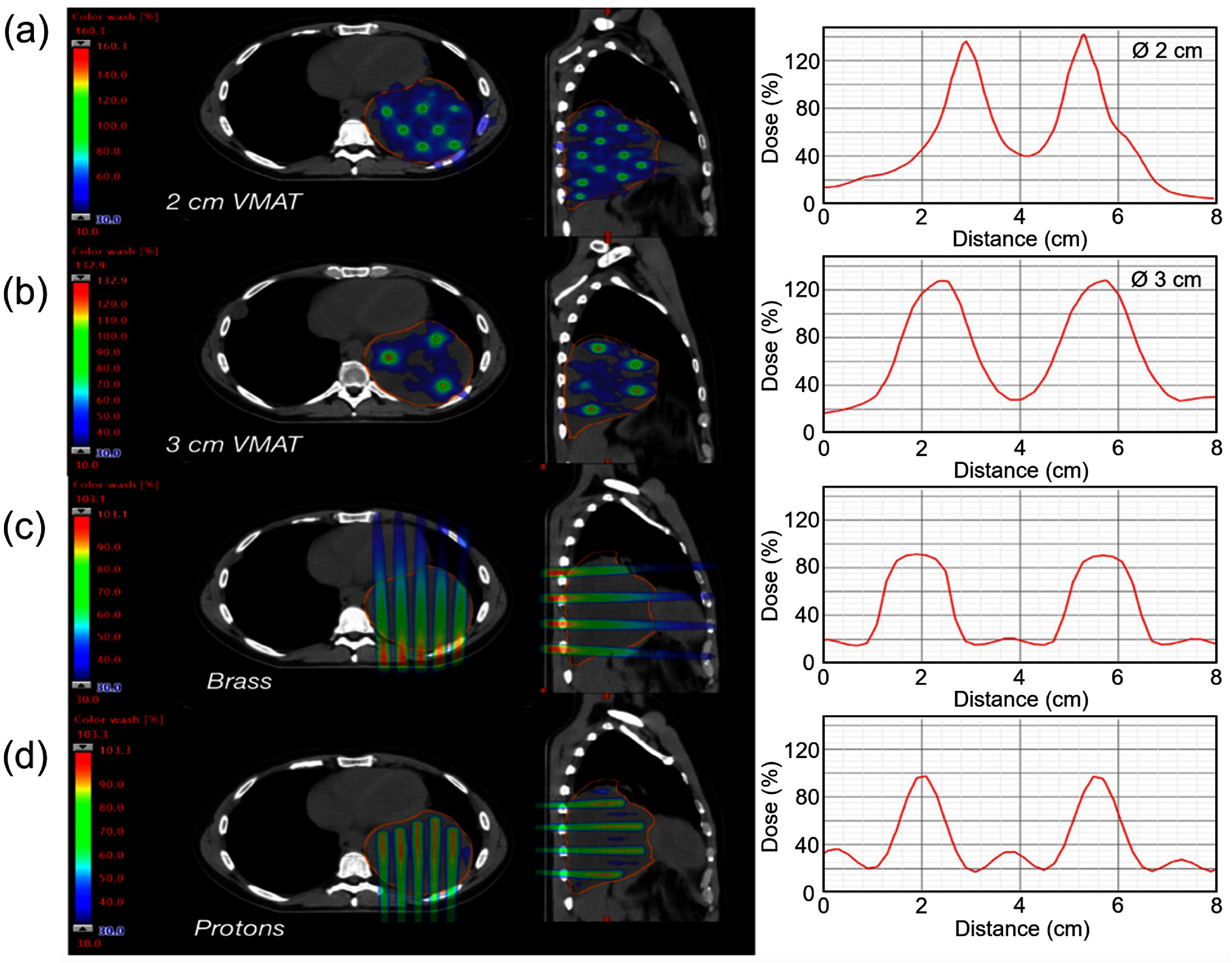
Examples of GRID and lattice RT for treating a bulky tumor in the thorax indicating axial and sagittal images with cross beam dose profile. Lattice RT using VMAT techniques with (a) 2 cm and (b) 3 cm center-to-center spacing. The sphere diameter (Ø) is 2 cm in (a) and 3 cm in (b). Larger CTC spacing provides higher PVDR and more flat areas in both peak and valley regions. (c) GRID therapy delivered by 6 MV photon beams using commercial brass collimator with divergent holes at CTC spacing of 2.1 cm in a hexagonal pattern and 1.4 cm peak width at isocenter. (d) GRID therapy delivered by proton beams using pencil beam scanning with the same CTC distance and aperture size of photon GRID shown in (c). Reprinted from [59], Copyright (2022), with permission from Elsevier.

Clinical implementations of SFRT include GRID RT and LRT [57]. These modalities differ in the spatial arrangement of their dose distributions. GRID RT typically involves a 2D pattern of peak and valley regions delivered via a single plane, while LRT employs a 3D array of high-dose vertices distributed within the tumor volume. Figure 6 illustrates representative dose distributions for various clinical SFRT techniques, demonstrating the characteristic peak-to-valley dose patterns with typical dose profiles.

#### 3.1.1. GRID RT.

GRID RT, introduced by Kohler in the early 1900s [37, 38], was designed to treat deep tumors with low-energy x-rays (∼200–400 kVp) while minimizing skin toxicity [60, 61]. Based on the idea that small tissue volumes could tolerate high doses, orthovoltage x-rays were used through grid collimators for over 50 years to treat various tumors. With the emergence of MV photon systems like ^60^Co and linacs, which offered better skin sparing, GRID RT using kV photons declined between the 1960s and 1990s due to its limitations in treating large tumors without excessive normal tissue damage [62]. In the 1990s, Mohiuddin *et al* revived GRID RT using MV photons for palliation in bulky tumors, typically delivering 10–20 Gy in one fraction through GRID blocks with 50% open areas [39, 63]. The adoption of multi-leaf collimators allowed 3D dose planning and parameter optimization, though it led to longer treatment times, higher surface doses, and dosimetric challenges from motion and small field effects [64, 65]. Proton GRID RT, especially with pencil beam scanning (PBS), offers better dose control and less normal tissue exposure than photons [66, 67]. Both MLC and PBS have expanded the clinical utility of GRID RT, now mainly used in palliative care for head and neck cancers, sarcomas, and large tumors, with >70% response rates [35, 68]. GRID RT is also used as a boost for enhancing outcomes in curable bulky tumors [69].

In GRID RT, the peak dose refers to the unblocked beam path, while the valley dose results from scatter and leakage [70]. The prescription dose is typically defined at the central peak, but valley dose and PVDR are also important [71]. There’s no consensus on optimal dosimetric guidelines, since these parameters vary with tissue depth, scattering, and beam geometry factors, such as beam divergence and penumbra [72]. In proton GRID RT, two strategies exist: using multiple fields to place the BP inside the tumor for a uniform dose distribution [47] or using a single field with the beam plateau region passing through the tumor for a non-uniform dose distribution [59]. Proton GRID shares similar field sizes with photon techniques, but PVDR is generally lower for deeper targets [66, 73]. A key advantage is the minimal exit dose due to the BP [73]. Other modalities, such as carbon ions [74] and very high-energy electrons (VHEEs, 100–250 MeV) [75], have also been explored. VHEEs offer favorable dosimetry but can generate significant bremsstrahlung radiation (x-rays that would lead to additional dose) from the collimators, typically made of high atomic number materials [76–78]. Using PBS with VHEEs can help reduce this effect and support clinical translation.

#### 3.1.2. LRT.

LRT, introduced in 2010, extends GRID RT by delivering high doses to discrete 3D regions (vertices) within a tumor, creating peak-to-valley dose distributions [79]. This spatial pattern resembles interstitial brachytherapy, with high-dose peaks surrounded by lower-dose valleys, enhancing tumor targeting while sparing nearby normal tissues [43]. LRT is especially useful for treating large, bulky tumors, often combined with conventional or hypofractionated CRT to achieve major tumor shrinkage (>50%) [79, 80], or used alone in an SBRT-like fashion [81, 82]. Three LRT design strategies exist: (1) geometric, using regular vertex placement [83]; (2) arbitrary vertex placement, for flexible planning; and (3) metabolism-guided, using PET/CT to place peaks in regions of high ^18^F-FDG uptake [84].

LRT has been implemented with MV photons and protons in treating gynecological, pulmonary, GI, and sarcomatoid tumors [79–83], using IMRT and VMAT techniques [85, 86]. Peak doses typically range from 5–25 Gy/fraction, with 50%–80% PVDRs and peripheral doses of about 3 Gy [43, 87], with smaller spacing used in boost treatments and larger spacing for standalone treatments [88–90]. Image guidance and motion management are essential to maintain accuracy of dose delivery.

### 3.2. Preclinical SFRT

Preclinical studies are usually designed to evaluate the safety, biological mechanisms, and therapeutic efficacy of a treatment approach, forming the foundation of clinical trial development. Although SFRT has been employed in clinical practice for over a century, many fundamental questions remain unanswered. Most preclinical SFRT investigations use murine or rodent models, which, due to their small size, require reduced radiation field dimensions and lower beam energies. Accordingly, preclinical SFRT research is generally classified into two main categories based on the beam size: MBRT and MRT [22].

#### 3.2.1. MBRT.

MBRT delivers spatially fractionated radiation with beam widths of 0.2–1.0 mm and CTC spacing from 0.8 mm to several millimeters, much smaller than in clinical GRID therapy. This enables high peak doses (up to 100 Gy) to tumors while minimizing damage to normal tissues [91, 92]. MBRT may offer advantages over conventional RT, particularly for radioresistant tumors, by exploiting the volume effect and stimulating regenerative responses in normal tissue [93–95]. Its precise dose distribution also makes it useful for studying SFRT’s biological mechanisms.

Most preclinical MBRT studies use kV x-rays with physical collimators on small animal irradiators [96–98]. These systems deliver 1–5 Gy min^−1^, with irradiation times from a few to over 20 min, raising concerns for motion-sensitive targets like lungs or abdomen [99]. Fixed collimator designs limit flexibility in studying optimal physical and geometric parameters, whereas modular collimators made from tungsten/brass and 3D-printed plastics enable customizable apertures [100]. New high-dose-rate irradiators (up to 125 Gy s^−1^) shorten treatment times, although uniform dose delivery over larger fields remains challenging [101]. Scanning MBRT using robotic stages offers an alternative, eliminating the need for multi-aperture collimators.

**Figure 7.**
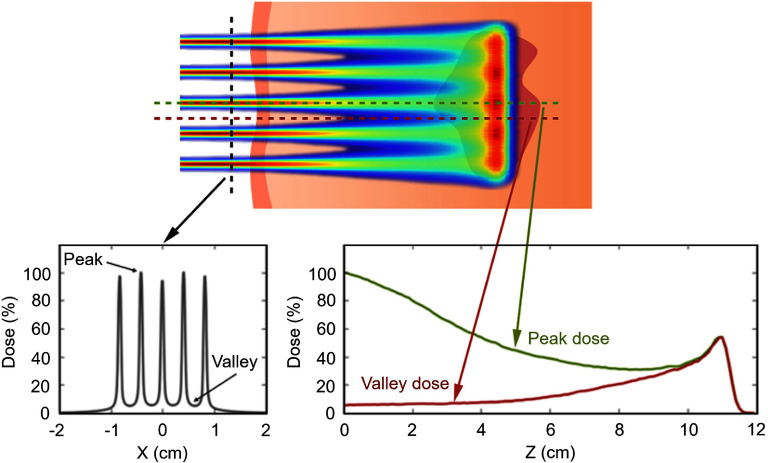
Illustration of proton MBRT delivered via pencil beam scanning to a deep-seated target, with cross-beam profiles shown at the surface and along peak and valley axes with depth. Due to multiple scattering of protons near the end of their range, lateral dose spread produces a relatively uniform dose distribution at the Bragg peak within the target. As depth increases, peak dose decreases and valley dose increases, leading to a reduced PVDR. Reprinted from [105], Copyright (2022), with permission from Elsevier.

Charged particles (e.g. protons, carbon ions) have also been used for MBRT, enabling narrow-beam scanning via magnetic fields without collimators [102–104]. Despite their benefits, dosimetry and radiobiological challenges remain, especially near the BP where scattering reduces PVDR [105]. As illustrated in figure 7, Proton MBRT achieves normal tissue fractionation while delivering a relatively uniform dose to deeper targets.

Clinical investigations include MBRT trials in dogs with brain tumors, comparing conventional stereotactic RT (3 × 9 Gy) to single-fraction MBRT (∼26 Gy mean dose with 6 MV x-rays). MBRT achieved ∼60% complete pathological response vs. partial remission in controls, with fewer long-term toxicities [106]. MBRT has also been used in humans for superficial tumors using 180 kVp x-rays, delivering 30 Gy in two fractions with 0.5 mm slits and 1.1 mm CTC spacing. Patients showed symptom relief and tumor response, with collimators fixed to minimize motion during 12 minute treatments [107].

#### 3.2.2. MRT.

Microbeam RT is a specialized type of RT that delivers high doses, ranging from hundreds to 1000 Gy, through extremely narrow beams (20–200 *μ*m wide), spaced 0.1–0.5 mm apart, using advanced synchrotron x-ray sources [108–111]. Unlike traditional GRID therapy or MBRT, MRT provides exceptional spatial resolution, making it an excellent tool for exploring biological responses to uneven radiation doses. First studied in the 1960s at Brookhaven National Laboratories for radiation safety and space research, MRT emerged as a potential therapy in the 1990s with synchrotron advancements [112]. Preclinical studies have demonstrated its ability to treat tumors, including brain, melanoma, and mammary cancers, while preserving healthy tissue, even at peak doses above 1000 Gy [113–117].

MRT is believed to affect biological systems differently from conventional RT, primarily through vascular damage, immune activation, and non-targeted effects (such as bystander and abscopal responses), rather than radiation-induced DNA damage [113–117]. MRT disrupts immature tumor vasculature while sparing normal vessels, enhances tumor vessel permeability (aiding drug delivery), and stimulates strong immune responses unlike conventional RT [118, 119]. Studies observed increased immune cell infiltration and pro-inflammatory gene expression, with *in-situ* vaccination effects and systemic tumor control [119–122].

In MRT, the CTC spacing is 4–8 times wider than the beam width, exposing more tissue to valley doses [123]. Low valley doses (<1 Gy) may enhance bystander and abscopal effects [124], while higher doses can suppress tumor regrowth and support immune and vascular responses. Studies have shown valley dose better predicts treatment efficacy than peak dose. Multi-angle beam delivery allows increased valley dose [125]. However, translating MRT’s advantages to larger-scale SFRT remains uncertain [113, 126]. Synchrotron x-rays enable millisecond-range delivery, crucial for avoiding motion artifacts in brain irradiation, where cardiac-induced movement (∼100–200 *µ*m) matches microbeam size [127, 128]. These UHDRs may also trigger the FLASH effect, reducing toxicity while preserving tumor control [129, 130]. However, ideal MRT parameters are still under investigation. Despite promising results, the optimal dosimetric and geometric parameters for MRT, specifically peak dose, valley dose, and beam spacing, for maximizing tumor control with reducing normal tissue toxicity remain unclear.

### 3.3. Challenges associated with SFRT

SFRT (GRID or LRT) combined with conventional RT has shown promising tumor and symptom control. Clinically, these techniques act as an up-front ‘boost’ to the uniform dose, taking advantage of valley-dose correlations with tumor response. Preclinical MBRT and MRT monotherapies also demonstrate strong control of aggressive, radioresistant tumors while sparing normal tissue. SFRT holds potential for tumors with poor local control under standard RT (e.g. glioblastoma, Ewing sarcoma) and settings where long-term toxicity is a concern (e.g. pediatric cancers, reirradiation). Targeting biologically distinct regions, such as areas of high metabolic activity identified on PET-SFRT, enables precise dose painting and may synergize with immunotherapies, as early clinical data show high symptomatic relief and occasional complete remissions [84].

Although significant progress has been made, further understanding is needed to fully harness SFRT for patient care. This requires addressing two key areas: (1) understanding radiobiology of SFRT, including immune activation, vascular effects, and bystander/abscopal mechanisms, as well as the basis for normal-tissue sparing; (2) correlating dosimetric/geometric parameters (peak width, valley dose, PVDR) with treatment outcomes [22]. Addressing these gaps requires both clinical trials that vary SFRT parameters systematically and preclinical studies that mimic clinical scenarios (e.g. SFRT followed by conventional RT or combined therapies). Bridging these areas will enable mechanism-driven dose prescriptions, SFRT-specific treatment planning tools, and broader clinician education.

## 4. FLASH RT

The conventional radiation doses are typically delivered at approximately 2–24 Gy min^−1^ (0.03–0.4 Gy s^−1^) average dose rate in mainstream practice of RT. For example, a 2 Gy fraction of a typical prescribed 60 Gy course of RT is delivered within several minutes depending on the intensity modulation. FLASH RT delivers doses at much higher average dose rates of ≳40 Gy s^−1^, and has attracted intense research interest in the past decade due to its potential to better spare healthy tissues while maintaining the same tumoricidal effect compared to RT at conventional dose rates [131–135]. Historical *in vitro* studies in the 1960s indicated that cell survival improved at UHDR (∼10^7^ Gy s^−1^ instantaneous dose rate) when the cells received the same dose of radiation at different dose rates [136, 137]. These early studies were only possible in research facilities for pre-clinical studies. In 2014, Favaudon *et al* demonstrated *in vivo* that mice receiving the same dose but at ultra high dose rates (>40 Gy s^−1^ average dose rate) resulted in less pulmonary fibrosis and coined the term FLASH RT [138].

The FLASH effect in RT refers to a biological phenomenon in which a reduction in normal tissues toxicity happens at UHDR while the damage to the tumor tissue remains the same [139]. The FLASH effect has been reported in various animal models and tissues by multiple investigators from different institutions around the world [23]. The exact mechanism responsible for the FLASH effect is yet to be understood [140]; however, several hypotheses have been proposed based on oxygen depletion [141], free radical reactions [142], mitochondrial damage [143], immunological response [144], and vascular effects [145]. The consensus is that the FLASH effect requires a minimum threshold for dose rate (≳40 Gy s^−1^) and dose (≳5 Gy). The clinical implementation of FLASH RT would significantly benefit from a deeper understanding of the physicochemical and biological mechanisms underlying the FLASH effect.

Safe and effective translation of FLASH RT requires stable radiation sources, reliable dosimeters, and understanding of the mechanism responsible for the FLASH effect. One important aspect of such studies to shed more light on the nature of the FLASH effect is the impact of the temporal structure of the radiation output (e.g. instantaneous dose rate, average dose rate, dose-per-pulse, etc). In conventional RT, the temporal structure of the radiation output is not relevant. However, in FLASH RT involving sub-second delivery of radiation, the instantaneous dose rate may play a role. The average dose rate is defined as ${\dot D_{\text{Avg}}} = \frac{D}{t}$, in which *D* is the total delivered dose over a time period *t*. However, for beams with distinct macro-pulses, the instantaneous dose rate or dose-rate-per-pulse is much higher than the average dose rate. The instantaneous dose rate is defined as ${\dot D_{{\text{Inst}}}} = \frac{D}{{N\tau }} = \frac{{{{\dot D}_{{\text{Avg}}}}}}{{f\tau }}$, where *N* is the number of delivered pulses, *f* is the pulse repetition frequency, and $\tau $ is the temporal pulse width [146].

**Figure 8.**
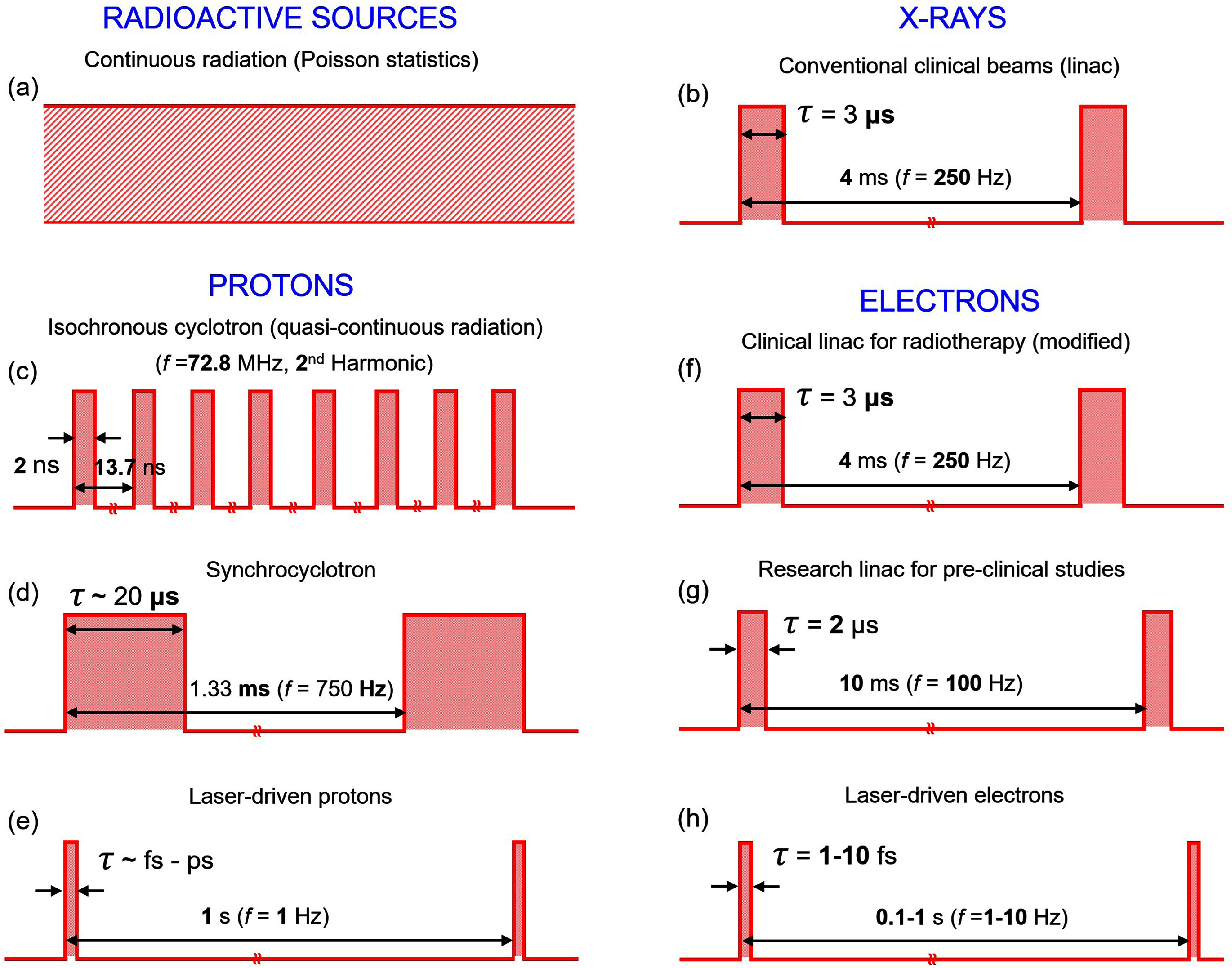
Temporal radiation output structure of a (a) radioactive source, (b) typical RT linac, (c) proton therapy isochronous cyclotron, (d) proton therapy synchrocyclotron, (e) laser-driven proton source, (f) modified clinical linac, (g) dedicated research linac, and (h) laser-driven electron source for UHDR research. [147] John Wiley & Sons. © 2020 American Association of Physicists in Medicine. Reproduced with permission from [146]. © 2022 The Authors. Medical Physics published by Wiley Periodicals LLC on behalf of American Association of Physicists in Medicine. CC BY-NC-ND 4.0. Reproduced from [148]. CC BY 4.0.

Figure 8 shows the radiation output structure of various radiation types. A radioactive source can be assumed to have a continuous radiation output (figure 8(a)); similarly, the output of an x-ray tube can be continuous radiation. However, in RT accelerators, the radiation consists of pulses, specifically micro- and macro-pulses. Figure 8(b) shows the output of a typical RT linac operating at 250 Hz with macro pulses separated 4 ms apart. Figure 8(c) corresponds to an isochronous cyclotron with a quasi-continuous output whereas in figure 8(d) a synchrocyclotron’s output is presented with distinct macro pulses separated 1.33 ms apart corresponding to 750 Hz. Figure 8(e) represents the output of a laser-driven proton source with ultrashort pulses (∼fs–ps) operating at 1 Hz. Figures 8(f) and (g) show the output of a typical RT linac modified for electron FLASH delivery as well as that of a dedicated pre-clinical research linac. Figure 8(h) shows the output of a laser-driven electron source with fs-scale pulses operating at 1–10 Hz.

### 4.1. Radiation sources

***Electrons***:

Developing radiation sources for FLASH RT is required for systematic studies. Conventional RT is primarily administered using MV x-ray beams typically with 6–18 MV energy. However, FLASH RT delivery of such photon energies is not achievable using exiting clinical linear accelerators due to the significantly low efficiency of the bremsstrahlung process. Most of the initial FLASH experiments have been performed using electron beams (∼4–10 MeV energy) produced by dedicated accelerators [149]. Later, it was shown that the conventional RT linear accelerators can be modified to deliver electrons at FLASH dose rates by retracting the x-ray target in a manner that high fluence of electrons are provided at the output. Placing the experimental subject (e.g. a mouse) at a close distance to the linac’s head allows a higher dose rate according to the inverse square law. However, typically, it will increase the dose rate by a factor of ∼2–3 which alone is not sufficient to reach FLASH dose rates (conventional RT dose rate is ∼2–24 Gy min^−1^ at 100 cm distance from the x-ray target). Further modification was done through applying a high beam current like the one used for photon beam delivery but with the x-ray target being retracted. As such, a high fluence rate of electrons will be achieved at the exit of the linac. Electron FLASH delivery with modified linacs has been shown by several investigators achieving average dose rates of several hundred Gy s^−1^ over a several centimeters radiation field size [150–152].

***Photons***:

Producing MV x-ray FLASH beams is challenging [129]. A few studies have been performed at national laboratories with synchrotron facilities [153]. It has been suggested that a multi-source accelerator simultaneously firing MV x-rays can achieve FLASH dose rates. However, such a system is associated with design complexities.

Kilovoltage x-ray FLASH on the other hand has been achieved over a small field size by bringing the subject as close as possible to the x-ray tube, taking advantage of the inverse square law. Cecchi *et al* demonstrated ∼100 Gy s^−1^ instantaneous dose rates over an ∼2 mm × 2 mm area in the proximity of an x-ray tube [154]. Rezaee *et al* demonstrate a dual-source cabinet-based x-ray FLASH irradiator for preclinical studies, providing dose rates around 100 Gy s^−1^ over a several cm^2^ field size [101, 155]. Tan *et al* through simulation proposed a multi-source FLASH irradiator composed of an array on miniature x-ray sources firing simultaneously, providing FLASH dose rates over a centimeter-scale area [156]. It should be mentioned that kilovoltage x-rays are only useful for small animal research or treatment of very superficial and small tumors.

***Protons***:

In the foreseeable future, proton FLASH RT is the most promising modality for human clinical trials allowing treatment of deeper targets [157]. Figure 2 shows the depth-dose curves of several beam types. Protons can penetrate deep in tissue and deliver the majority of the dose at a specific location known as the BP. There are three main technologies to produce protons of therapeutic energies: isochronous cyclotron, synchrocyclotron, and synchrotron. FLASH delivery has been demonstrated using all these modalities by increasing the initial beam current and further optimization and tuning of some operating parameters [147, 158–160]. Human clinical trials are ongoing using proton FLAHS RT [161]. Proton FLASH RT has been studied through shoot-through or transmission of conformal FLASH [162, 163].

The state-of-the-art proton therapy systems employ a technique known as PBS to expand the radiation field in a plane perpendicular to the radiation beam. In order to cover the extent of the tumor along the beam direction, multiple energies are used so that the whole tumor volume receives the prescribed dose. In the context of FLASH RT, PBS would complicate the dose rate measurement as each voxel in the target may not receive the same dose rate. As such, various definitions have been proposed to define the dose rate in scanning fields [164], such as PBS dose rate [165], dose-averaged dose rate [166], and average dose rate [158]. Measuring such dose rates in 3D requires dedicated dosimeters with high spatiotemporal resolution.

Static proton fields produced through passive scattering techniques, which now have been replaced by PBS systems, provide more uniform dose rate distribution across the volume. However, achieving a FLASH dose rate using passive element for field shaping is not practical for large fields. Nevertheless, static beam shaping through passive elements is appealing for small animal experiments dealing with small fields (∼1 cm^3^). Various designs have been demonstrated to achieve such fields [167].

### 4.2. FLASH dosimetry

Accurate measurement of the radiation dose is of utmost importance in RT QA. A variety of active and passive, single- and multi-element dosimeters, as well as dosimetry protocols (e.g. AAPM’s TG-51 [168], TG-61 [169], and TRS 398 [170]) exist for conventional RT machines. Table 2 summarizes a list of dosimeters with their features. FLASH RT dosimetry is not straightforward due to the UHDRs involved. Significant research has been devoted to developing dedicated dosimeters and dosimetry techniques for FLASH RT [146, 171, 172].

**Table 2.** Characteristics of RT dosimeters. Reproduced from [148].

Dosimeter	Reference dosimetry	Beam monitor	Spatial resolution	Real time	Temporal resolution	Accuracy	2D	*In vivo*	Water-equivalent
Ion chamber	Yes	Yes	Several mm	Yes	100–200 *µ*s	1%–2%	Array	No	∼Yes
Calorimeter	Yes	Yes	∼ cm	Yes	ms–10 s ms	1%	No	No	Yes (water)
Fricke	Yes	No	Sub-mm to cm	No	N/A	1%	Potentially	No	∼Yes
Semiconductor	No	Yes	Sub-mm	Yes	1–10 ns	2%–5%	Array	Yes	No
RC Film	No	No	Sub-mm	No	N/A	3%–5%	Yes	Yes	∼Yes
TLD	No	No	Several mm	No	N/A	3%–10%	No	Yes	∼Yes
OSLD	No	No	mm (sub-mm)	No	N/A	3%–5%	Array	Yes	No
RL dosimeter (Scintillator)	No	Yes	Sub-mm to several cm	Yes	Ns–*µ*s	3%–5%	Yes (Array)	Yes	∼Yes (organic)

Ionization chambers are the gold standard in RT dosimetry and the recommended device for RT machine calibration [173]. Commercial devices based on multilayer ion chambers are available to provide 2D measurements in a plane perpendicular to the beam or along the beam. Due to UHDRs involved in FLASH beams, ion recombination in these devices can impact the measurement if not properly mitigated [158]. It has been shown that increasing the operating potential or reducing the separation between the electrodes can mitigate the impact of ion recombination. Furthermore, several prototypes of multilayer ionization chambers have been designed and are under development to perform 2D and 3D dosimetry in FLASH beams [174–176].

Semiconductor-based devices are an attractive choice in RT dosimetry due to their higher sensitivity allowing for high resolution and multi-dimensional dosimetry. In an ion chamber ∼34 eV is needed to produce an ion-pair, whereas in a silicon ∼3.4 eV is required to produce an ion-pair [177]. Due to their crystalline nature, they suffer from orientation dependency [12]. They also suffer from dose rate dependency [178]. Silicon is not water equivalent; however, synthetic diamonds have an effective atomic number closer to that of water. Researchers are working on developing diamond detectors for FLASH dosimetry and beam monitoring [179].

Radioluminescent dosimeters are attractive choices for RT dosimetry; they can provide high spatiotemporal resolution multi-dimensional measurements [148, 180]. Plastic scintillators have water-equivalent properties. Scintillation-based dosimeters, when synchronized with the accelerator’s pulse train, can provide pulse-by-pulse measurement [181, 182]. Scintillators coupled with optical fibers can provide point measurement with high resolution. Cherenkov radiation and ionization quenching are two main challenges associated with scintillation dosimetry [183, 184]. The former is a significant issue in photon and electron fields, while the latter is an important issue in ion beams such as protons [185, 186]. While Cherenkov light is a parasitic signal in scintillation dosimetry, it should be noted that imaging the Cherenkov light in undoped media, such as water or patient’s skin, can be used for relative dosimetry in electron and photon fields [187]. Researchers are working on developing radioluminescent-based dosimeters with high spatiotemporal resolution for FLASH RT.

Calorimetry provides absolute measurement of the radiation dose by measuring the induced heat in the dosimeter material, typically graphite or water [12]. Water calorimeters are bulky and used in standard laboratories. However, graphite-based calorimeters have been used for FLASH RT dosimetry [171]. The spatial resolution is limited, and they may not be optimal for small-field dosimetry. Researchers are working on developing calorimeters with high temporal resolution to measure the temporal dynamics of beams.

Radiochromic film dosimeters are composed of radiosensitive monomers that undergo solid-state polymerization upon irradiation [188, 189]. The radiation-induced polymerization, manifested as a visible darkening of the films, is proportional to the dose and is quantified by the optical density [190, 191]. It has been shown that radiochromic films can be used for UHDR dosimetry [146]. However, caution must be exercised in using film for proton therapy dosimetry as their response has been shown to be impacted by the linear energy transfer (LET) of the beam [192, 193]. Other passive dosimeters, such as TLDs and OSLDs have been found to be appropriate for FLASH dosimetry [194].

### 4.3. Pre-clinical FLASH RT

Preclinical research over the last decade across multiple animal models, biological tissues, and beam modalities has produced a growing body of literature pointing toward the potential of FLASH RT to preserve normal tissue while maintaining tumor control, the so-called FLASH effect. In rodent models of the brain, FLASH preserved neurocognitive function, reduced neuroinflammation, and mitigated radiation-induced cognitive decline compared with RT at conventional dose rates [153, 195]. Similar protective effects were observed in the lung and thorax, where FLASH delivery reduced acute pulmonary fibrosis and long-term lung injury without compromising tumor response [196–198]. Experiments in both small animals and pigs confirmed reduced skin toxicity [199], while abdominal irradiation studies showed mixed results, with some reporting gastrointestinal sparing [200] and others showing no protective effect [201]. Importantly, FLASH RT achieved tumor control outcomes comparable to conventional RT across diverse tumor models, including glioma, lung, melanoma, and breast cancer, with studies reporting similar tumor growth delay, local control, and survival. These findings have been generated using different beam modalities: mostly electrons, which dominate small-animal research; protons, where preclinical evidence is rapidly expanding and shows promising tissue-sparing effects; and, to a lesser extent, photons, which nonetheless support the presence of normal tissue protection under FLASH conditions [202]. Recent studies using orthovoltage x-rays with high-power rotating-anode sources further demonstrated the feasibility of preclinical photon FLASH. Miles *et al* showed that ocular irradiation in mice preserved retinal function and spared inner retinal damage at FLASH dose rates compared with conventional irradiation [203]. In another investigation, the same group reported reduced radiation-induced skin injury while maintaining tumor growth suppression in mice irradiated with x-ray FLASH, thereby confirming normal tissue protection without loss of tumor control [204].

Consensus in preclinical FLASH RT research suggests that a dose rate of at least ∼40 Gy s^−1^ is generally required to induce the FLASH effect, and this must be paired with a sufficiently high single-fraction dose (greater than ∼5 Gy). Carlier *et al* indicated that most preclinical FLASH protocols assume a minimum dose of ∼4–10 Gy for normal-tissue protection [205]. An *in vitro* study showed that when a single dose reached 20 Gy, FLASH RT reduced DNA damage in lung fibroblasts and increased the cell survival rate and when a single dose was <20 Gy, the protective effect of FLASH RT disappeared [206]. These findings suggest that the threshold dose for inducing the FLASH effect is organ-specific and falls within a window for each tissue. Van Marlen *et al* modeled FLASH scenarios using thresholds of both ∼4 and ∼8 Gy, alongside the 40 Gy s^−1^ dose rate, to assess clinical feasibility [207]. Their analysis, performed in the context of SBRT (34 Gy, single-fraction lung SBRT), showed that transmission beam plans could theoretically achieve dose rates high enough for FLASH RT, with FLASH-percentages ranging from ∼30%–80% depending on beam delivery parameters. Importantly, they found that optimizing machine factors (e.g. shorter minimum spot times, higher nozzle currents, and spot-based gantry current techniques) increased the achievable FLASH dose percentage. However, despite technically feasible dose rates, the authors concluded that the *dose threshold* is likely the primary limiting factor in clinical FLASH realization, rather than the ability to achieve UHDRs.

A key trend from preclinical studies is that beam parameters strongly influence biological outcomes. A pooled meta-analysis of 41 experiments quantified tumor control and normal tissue sparing into a Therapeutic Index Score and correlated this with beam features [208]. The strongest associations were with pulse dose rate (*r* = 0.491, *p* = 0.038) and pulse dose (*r* = 0.476, *p* = 0.046), suggesting that short, high-dose pulses improve the therapeutic index. Mean dose rate correlated with normal tissue sparing, while total dose correlated with tumor control. These results emphasize the importance of both temporal structure and absolute dose, though the authors caution that study heterogeneity and semi-quantitative scoring may limit interpretation.

Large-animal studies in companion dogs have become an important bridge between rodent models and human translation of FLASH RT. At the University of Pennsylvania, researchers have launched a series of clinical trials using proton FLASH in dogs with naturally occurring cancers, including extremity sarcomas [209]. These efforts are part of a broader NIH-funded program on particle FLASH and represent some of the first systematic investigations of proton FLASH RT in veterinary oncology. In Europe, Børresen *et al* reported outcomes from a prospective cohort of privately owned dogs with macroscopic oral cancers treated with single-fraction electron FLASH at doses ⩾30 Gy [210]. Their study demonstrated promising tumor control but also revealed significant late toxicities, including osteoradionecrosis, underscoring the challenges of applying single high doses in the oral cavity. A follow-up analysis by Gjaldbæk *et al* confirmed the anti-tumor efficacy of electron FLASH RT in canine oral and nasal cancers [211]. Also, it pointed out that late bone and mucosal toxicity becomes a limiting factor around 30 Gy, suggesting the need for more refined dose and fractionation schemes. Beyond the University of Pennsylvania and the Scandinavian cohorts, veterinary clinical trial registries show ongoing proton FLASH trials in dogs with sarcomas and osteosarcomas, reinforcing the growing translational focus on large-animal models. In parallel, the University of Wisconsin–Madison has initiated a trial of FLASH RT in pet dogs with osteosarcoma, illustrating that multiple academic veterinary centers are now contributing to this field [212]. Collectively, these canine studies feature both the promise of FLASH RT to improve the therapeutic index in clinically relevant settings and the challenges of balancing tumor control with site-specific late toxicities.

### 4.4. Clinical trials of FLASH RT

In 2019, Lausanne University Hospital conducted the first human clinical trial of electron beam FLASH RT [213]. They treated a 3.5 cm cutaneous T-cell lymphoma lesion with a single 15 Gy FLASH electron fraction (90 ms delivery). The only acute skin findings were grade-1 epithelitis and transient grade-1 oedema, which the authors state peaked at approximately 3 weeks post-treatment; clinical exam and optical coherence tomography showed no epidermal thinning or basal-membrane disruption. The case report describes these reactions as transient and documents a durable complete tumor response at 5 months, but it does not specify the exact date when the grade-1 skin changes fully resolved.

In 2020, Cincinnati Children’s Hospital Medical Center/University of Cincinnati Health performed the first proton FLASH RT trial (FAST-01, NCT04592887) [214] on 10 patients (aged 27–81) with painful bone metastases, aimed at assessing the safety of proton FLASH beam delivery. Twelve lesions received a single 8 Gy dose at 60 Gy s^−1^ of shoot-through protons. The actual irradiation time was under 1 s. Three months post-treatment, seven patients achieved complete or partial remission. Pain relief was comparable to conventional palliative RT, and side effects were mild and transient, mainly mild skin pigmentation (Grade 1). These results demonstrate the safety and feasibility of proton FLASH RT. Building on these findings, the University of Cincinnati and Varian Medical Systems initiated the FAST-02 clinical trial targeting painful bone metastases in the thoracic region [215]; the study enrolled 10 participants and focused on treatment-related side effects and pain relief outcomes. While completion of enrollment was recently announced, the FAST-02 trial is still ongoing.

### 4.5. Challenges associated with FLASH RT

FLASH RT shows great potential for sparing healthy tissue while effectively treating tumors, but its clinical translation is not straightforward. Conventional dosimeters may have sub-optimal performance under UHDR beams, leading to inaccurate dosimetry. Beam structure variability across machines further complicates FLASH dosimetry and dose rate description. Real-time beam monitoring remains challenging, requiring detectors with high spatiotemporal resolution, transparency, and fast feedback. A wide dynamic range is also needed to be compatible at both UHDR and conventional RT dose rates. Biological uncertainties on the FLASH effect and optimum dose and dose rate add to its complexity. Addressing these technical and biological issues is essential for clinical translation of FLASH RT.

Electron FLASH RT has demonstrated early success in treating superficial tumors using dedicated devices [216]. Proton FLASH RT enables deep-seated tumor treatment at UHDRs, but beam shaping to conform to larger tumors while maintaining the UHDR is challenging. VHEEs (>100 MeV) offer promising penetration depths (5–30 cm), uniform dose distribution, and reduced scattering [217, 218], although their normal tissue-sparing effects remain under investigation [219]. Photon-based FLASH RT also faces challenges due to low conversion efficiency of the bremsstrahlung process and hardware limitations [220]. Understanding the responsible radiobiological mechanisms, clinical translation of FLASH RT, particularly for deep tumors, depends on advancements in beam delivery, accurate dosimetry, and the development of compact and cost-efficient systems [221].

Although preclinical reports of FLASH-mediated normal-tissue sparing span species and modalities, there is substantial heterogeneity in outcomes that must be acknowledged. Experimental and beam parameters, especially dose-per-pulse, dose-rate-per-pulse, average dose rate, total dose, and irradiation time, strongly influence whether a FLASH benefit is observed, and threshold effects vary between tissues and models [208]. Reproducibility has proven sensitive to differences in accelerator output, beam structure, dosimetry methods, and incomplete reporting, which complicates cross-study comparisons. Biological variables, such as tissue oxygenation and model-specific radiosensitivity, also modulate the response. Encouragingly, multi-institutional studies have demonstrated that with harmonized beam settings and rigorous protocols, the normal tissue sparing effect can be consistently reproduced across laboratories [222]. Nonetheless, conventional dosimeters struggle under UHDR conditions; there is a lack of agreed dosimetry standards, and existing devices may under-respond or misreport doses, emphasizing the need for new dosimeters and real-time beam monitoring systems [146]. Structured acceptance, commissioning, and QA frameworks covering essential parameters such as pulse structure and dose-per-pulse are being developed to ensure cross-platform consistency and will pave the way for robust clinical protocols [223].

## 5. NPs in RT

NPs have gained attention in RT due to their small size, biocompatibility, and potential in targeting tumors via both passive mechanisms, such as the EPR effect [25, 224, 225], and active targeting through ligand or antibody conjugation [24]. High-atomic-number (high-Z) metal NPs, like gold (AuNPs) and hafnium oxide (HfO_2_), act as effective radiosensitizers by enhancing local dose absorption via the photoelectric effect and emission of Auger electrons [226–228]. Their high surface area supports drug loading and contributes to phenomena like surface plasmon resonance, enabling strong optical responses for imaging and therapy [229].

**Figure 9.**
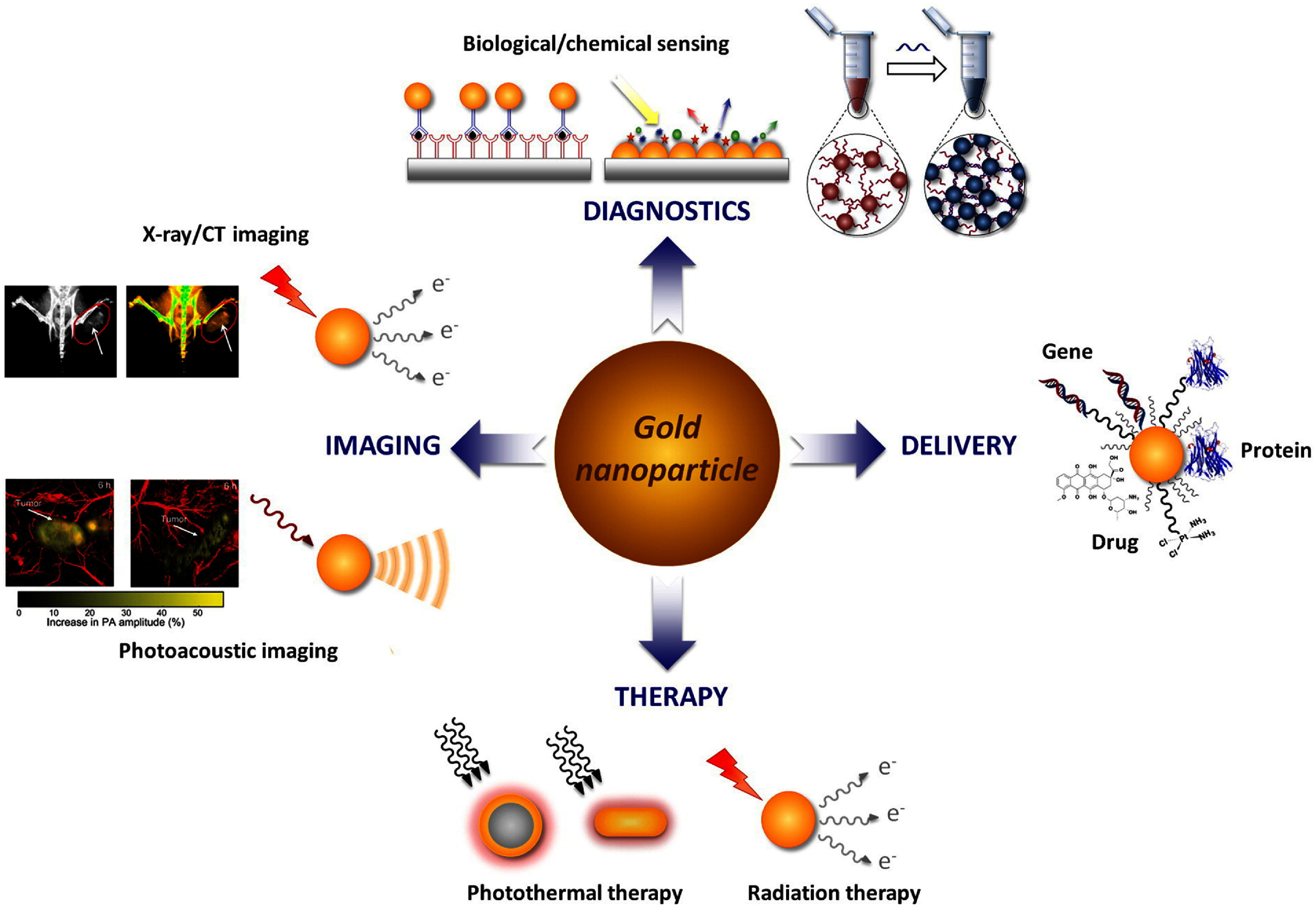
Biomedical Applications of AuNPs. Reprinted from [229], Copyright (2017), with permission from Elsevier.

Other metal and metal oxide NPs, such as palladium (PdNPs), platinum (PtNPs), silver (AgNPs), and zinc oxide (ZnONPs), also show promise in NP-enhanced RT due to their tunable physicochemical and optical properties [230–232]. AuNPs are particularly versatile due to their customizable synthesis and unique electronic and optical features [26, 229] enabling a wide range of biomedical applications (figure 9): Imaging: CT, photoacoustic imaging, and surface-enhanced Raman scattering; Delivery: Drugs, genes, and siRNAs; Therapy: Photothermal therapy and radiosensitization; Diagnostics: Biological and chemical sensing platforms.

### 5.1. Radiosensitization by high-Z elements

The photon interaction mechanisms that dominate when materials are irradiated with x-rays are Rayleigh scattering, photoelectric effect, Compton scattering, and pair production [12]. The photoelectric effect is highly relevant to radiosensitization, as it ejects inner-shell electrons whose energy can only damage nearby tissues due to their limited range. Its cross section is approximately proportional to (*Z/E*)^3^, where *Z* is atomic number of the material and *E* is photon energy. The relaxation of these ionized atoms produces fluorescence photons or Auger electrons, the latter of which travel only ∼10 nm causing dense, localized DNA damage which is effective only if it occurs near target molecules. Compton scattering, in contrast, is independent of the atomic number and results in lower-energy scattered photons and ejected electrons. High-*Z* NPs primarily enhance radiation dose through photoelectric interactions [233–236].

This principle underlies the use of high-*Z* metal NPs (e.g. gold) as radiosensitizers to increase tumor-localized radiation effects. Early dose enhancement was noted in contrast agent imaging studies [237], where iodine and gadolinium were used under kV and MV irradiation. However, AuNPs, with higher *Z* and better tumor targeting, offer greater radiosensitization. Preclinical animal studies using 250 kVp x-rays showed that AuNPs significantly improved tumor regression and survival [233, 238], and were particularly effective in treating radioresistant tumors like squamous cell carcinoma [239].

NPs enhance RT efficacy through multiple synergistic mechanisms. Upon irradiation, they amplify reactive oxygen species (ROS) production, inducing oxidative stress that induces damage to DNA, proteins, and lipids, leading to adverse biological effects such as apoptosis [240, 241]. By localizing within tumors, NPs intensify double-strand DNA breaks, the most lethal form of damage, thereby increasing tumor specificity and minimizing harm to healthy tissue [242, 243]. They also help overcome hypoxia by improving local oxygenation, making resistant regions more radiosensitive [244]. Additionally, NPs can arrest cancer cells in the G2/M phase, where cells are most vulnerable to radiation, enhancing cytotoxic effects [245, 246]. Together, these effects significantly boost the therapeutic efficacy of NP-enhanced RT.

### 5.2. Preclinical applications of NPs in RT

Preclinical studies have extensively explored NPs as radiosensitizers to enhance the efficacy of RT in cancer treatment, leveraging their ability to amplify radiation effects through physical, chemical, and biological mechanisms. High-*Z* elements, such as gold (*Z*= 79), bismuth (*Z* = 83), gadolinium (*Z* = 64), and hafnium (*Z* = 72), are commonly incorporated into NPs to increase x-ray absorption, leading to enhanced production of secondary electrons, ROS, DNA damage, and cell cycle arrest, ultimately promoting tumor cell death [247]. For example, Hainfeld *et al* demonstrated that intravenous administration of 1.9 nm gold NPs in mice with subcutaneous EMT-6 mammary tumors, combined with 250 kVp x-ray irradiation, resulted in an 86% 1 year survival rate compared to only 20% with radiation alone [238]. Similarly, gadolinium-based polysiloxane NPs, known as AGuIX, significantly improved survival in preclinical models, including rats with 9L gliosarcomas and mice with melanoma. In gliosarcoma models, treated animals survived on average 72.9 d versus 39 d with radiation alone [248–250]. Hafnium oxide nanoparticles (NBTXR3) have also shown impressive results: Maggiorella *et al* reported an 82% inhibition of tumor growth in fibrosarcoma xenografts following intratumoral injection and irradiation [251].

In addition to inorganic NPs, drug-loaded formulations have demonstrated radiosensitizing potential. For instance, docetaxel (DOC), a chemotherapeutic agent with radiosensitizing properties, shows limited efficacy on its own. A study developed gelatinase-responsive docetaxel-loaded NPs (DOC-NPs) and found that they significantly enhanced radiosensitivity in gelatinase-overexpressing gastric cancer cells, while sparing normal gastric cells [252]. Overall, these studies underline the ability of NPs to improve tumor targeting through mechanisms such as EPR, active targeting, or local administration, while reducing collateral damage to healthy tissues. Such findings support the potential for clinical translation of NP-based radiosensitizers in cancer therapy.

### 5.3. Clinical trials of NPs in RT

Building on preclinical successes, NPs have begun to show promise as radiosensitizers in clinical settings. Early trials have primarily focused on safety, tolerability, and efficacy in enhancing RT outcomes, particularly using formulations such as AGuIX (gadolinium-based) and NBTXR3 (hafnium oxide). AGuIX serves both as a radiosensitizer and an MRI contrast agent. Phase 1/2 trials in brain metastases and glioblastoma have shown good tolerability and potential for improved local tumor control. In a phase 1b trial (NCT04094077), AGuIX combined with stereotactic radiotherapy in patients with oligo brain metastases was well tolerated, showing encouraging tumor response [253]. In glioblastoma, the NANO-GBM Phase 1b trial evaluated intravenous AGuIX NPs alongside standard radiotherapy (60 Gy) and temozolomide (TMZ) in eight patients with partially resected tumors. Patients received four AGuIX injections with concomitant and adjuvant TMZ. Only one dose-limiting toxicity occurred (grade 3 lymphopenia, related to TMZ), and no severe toxicities were attributed to AGuIX. MRI imaging confirmed selective accumulation in tumor tissue, and the recommended Phase 2 dose (RP2D) of 100 mg kg^−1^ was well tolerated, supporting further clinical development [254].

NBTXR3, a hafnium oxide NP activated by RT to induce localized electron emission and tumor cell death, has been evaluated in multiple cancers, including head and neck squamous cell carcinoma (HNSCC), lung cancer, and soft tissue sarcomas. In a phase 1 trial for locally advanced HNSCC (NCT01946867), intratumoral NBTXR3 combined with radiation led to significant tumor shrinkage while preserving function in elderly or comorbid patients, with manageable side effects [255]. Additional phase 1/2 studies, such as NCT04505267 in inoperable lung cancer and NCT03589339 combining NBTXR3 with anti-PD-1 immunotherapy, demonstrated enhanced tumor destruction without increasing toxicity to surrounding tissues, translating into improved response rates and overall survival [256–259].

Overall, these clinical studies underscore the potential of NPs to overcome radioresistance, enhance localized radiation effects, and allow combination with systemic therapies. Ongoing trials, including some approaching phase 3, are investigating dose optimization and broader applicability, although long-term outcomes and wider clinical translation remain areas for future research.

### 5.4. NPs in SFRT

Combining NPs with MRT at UHDR has transformative potential to improve therapeutic outcomes through enhancing tumor tissue damage while sparing healthy tissues. Early studies used Monte Carlo (MC) simulations to explore the effects of beam and array parameters on biological endpoints such as DNA damage [40, 260, 261]. Gadolinium (Gd) NPs showed dose enhancement in kV beams, especially at 60–70 keV [262]. MC tools like Geant4-DNA, EGSnrc, and TOPAS have since modeled NP effects on dose and DNA damage with reasonable experimental validation [258, 259, 263–265]. MC studies have shown that larger gold NPs (100 nm) and clustering near the nucleus significantly enhanced DNA damage due to increased secondary electron production [263]. Gadolinium NPs produce higher-energy electrons with longer range, and clustering affects dose distribution, though radiosensitization remains effective as predicted by LEM models [266]. Iron-gold heterojunctions with ⩾50% gold content yield stronger DNA damage, especially at low photon energies (∼50 keV), due to enhanced photoelectric effects [266]. Overall, MC simulation is a useful tool for optimizing NP-enhanced MRT, offering insight into dose distribution, NP behavior, and biological effects. These tools can guide future clinical translation. Nevertheless, MC simulation packages still lack accurate models for many fundamental processes in biological systems.

Preclinical studies have shown that MRT delivers high doses to tumors with minimal toxicity to normal tissues in rodents, piglets, and dogs [267–270]. NP integration may further enhance MRT efficacy. Gadolinium-based nanoparticles (GBNs) serve as dual MRI contrast agents and radiosensitizers, improving tumor targeting and survival in rats [271–273]. Theranostic NPs combining gadolinium and bismuth are compatible with CT/MRI-guided RT [272]. Recently, gel dosimeters with silver nitrate were also developed for accurate dose measurement in synchrotron MRT/FLASH RT [274]. Studies using AuNPs, GBNs, and Ta_2_O_5_ show enhanced dose delivery, DNA damage, and tumor control, with minimal effects on normal tissue [275–279]. AuNPs accelerated endothelial cell migration and enhanced radiosensitization, especially at 40 keV photon energy [276, 277]. Ta_2_O_5_ showed selective cytotoxicity in tumor vs. normal cells under synchrotron beams [278]. In DU145 and A549 cells, AuNPs increased morphological damage post-irradiation, with no effect on normal cells, suggesting selective radiosensitization [278, 279]. GNPs significantly increased gliosarcoma cell sensitivity to 125–150 kVp x-rays, with a SER of 1.43 at 500 *μ*g ml^−1^ [280]. Together, these studies support MRT combined with NPs as a promising strategy for improved tumor control with reduced side effects.

### 5.5. Challenges associated with NPs in RT

NPs offer promising enhancements in RT by improving tumor targeting and increasing radiosensitivity, but several interrelated challenges hinder their clinical translation. Ensuring biocompatibility and long-term safety is critical, since some metallic NPs (e.g. gold, silver) can accumulate in organs and may induce oxidative stress or other forms of cellular damage, raising toxicity concerns [281]. At the same time, achieving uniform NP distribution within tumors is difficult: irregular tumor vasculature, elevated interstitial pressure, and variable lymphatic drainage produce heterogeneous intratumoral NP accumulation, which reduces predictability of radiosensitization and complicates treatment outcomes [282]. Manufacturing and regulatory hurdles further impede progress, such as scaling up production while maintaining strict quality, batch-to-batch consistency, and demonstrating safety/efficacy through extensive clinical testing remains complex and costly [283].

The heterogeneity of the EPR effect across tumor types, within lesions, and between patients means that reliance on passive accumulation alone is often insufficient [284]. To improve reproducibility and clinical impact, multiple complementary strategies are under active investigation: (1) rational engineering of NP physicochemical properties (size, shape, surface chemistry and charge) to enhance penetration and retention [285]; (2) active targeting and stimuli-responsive systems (ligands, antibodies, pH/enzymatic or externally triggered release) to increase tumor selectivity [285]; (3) theranostic and image-guided approaches to select patients and lesions with favorable NP uptake in real time (for example, NPs that provide MRI/CT contrast) [286]; and (4) local delivery strategies such as intratumoral injection when systemic EPR is inadequate, an approach already used in clinical studies of locally delivered NP radiosensitizers [287]. Taken together, these avenues, supported by careful preclinical modeling, imaging-led patient selection, and manufacturing standardization, are essential to reduce real-world variability and to enable reproducible, safe integration of NPs into radiotherapy protocols.

## 6. Summary

SFRT, FLASH RT, and NP‐enhanced RT are techniques under development and active research with potential to spare healthy tissues and improve the effectiveness of RT. Combining them may further enhance their benefits for RT, resulting in an additive or synergistic effect. SFRT delivers non-uniform dose distribution, creating low-dose valley and high-dose peaks, which activate immunological and biological responses other than proliferative cell death induced by conventional RT techniques. FLASH RT delivers UHDR (⩾40 Gy s^−1^), triggering the ‘FLASH effect’ that reduces normal‐tissue damage. NP‐enhanced RT relies on high‐Z NPs that accumulate in tumors and locally boost dose through generation of high-LET secondary electron emission. When NPs are loaded into SFRT‐targeted areas and irradiated at FLASH dose rates, one may achieve a focused, tumor‐specific dose boost with multiple protective and sensitizing mechanisms working together.

Technologically, SFRT requires high-resolution and high dose rate beams, while FLASH RT needs dedicated accelerators capable of microsecond‐range and ultra‐high‐current pulses. NP integration demands controlled synthesis (size, surface chemistry, targeting ligands) to ensure uniform tumor uptake. Dosimetry is especially challenging: SFRT’s steep dose gradients call for submillimeter detectors (e.g. radiochromic films, microdiamond probes), while FLASH’s extreme dose rates require ultra‐fast detectors. Treatment‐planning systems may incorporate MC models that account for NP‐induced dose enhancements at the nanoscale.

Biologically, SFRT’s spatial heterogeneity might trigger vascular and immunogenic effects; FLASH RT could spare normal cells by modifying free radical production and rapidly depleting oxygen, though exact pathways remain under study; and NP-RT may intensify local DNA damage and possibly modulate the tumor microenvironment. Clinical translation may rely on an interdisciplinary team, including applied and medical physicists, engineers, radiation biologists, material scientists, chemists, and oncologists, to develop and refine the technology of the irradiation systems, standardize dosimetry, optimize NP design, and develop early‐phase trials. An initial focus could be on the combination of SFRT with NP-enhanced RT, building on early studies on MRT and NP, with progressive incorporation of dose-rate effects, including UHDR. Research is ongoing toward developing dedicated radiation sources and beam modifiers, dosimeters, as well as novel NPs to further explore the biological mechanisms of these modalities.

## Data Availability

All data that support the findings of this study are included within the article (and any supplementary files).
